# Vesicular dysfunction and pathways to neurodegeneration

**DOI:** 10.1042/EBC20210034

**Published:** 2021-12-22

**Authors:** Patrick A. Lewis

**Affiliations:** 1 Department of Comparative Biomedical Sciences, Royal Veterinary College, University of London, London, United Kingdom; 2 Department of Neurodegenerative Disease, UCL Queen Square Institute of Neurology, London, United Kingdom; 3 Aligning Science Across Parkinson's (ASAP) Collaborative Research Network, Chevy Chase, MD, United States of America

**Keywords:** axonal transport, endocytosis, mitophagy, neurodegeneration, trafficking

## Abstract

Cellular control of vesicle biology and trafficking is critical for cell viability, with disruption of these pathways within the cells of the central nervous system resulting in neurodegeneration and disease. The past two decades have provided important insights into both the genetic and biological links between vesicle trafficking and neurodegeneration. In this essay, the pathways that have emerged as being critical for neuronal survival in the human brain will be discussed – illustrating the diversity of proteins and cellular events with three molecular case studies drawn from different neurological diseases.

## Introduction

Neurodegenerative diseases, characterised by the progressive loss of neurons in the central nervous system, impact on the lives of millions of people around the world [1]. These disorders span a range of clinical presentations, from disorders characterised primarily by cognitive dysfunction such as Alzheimer's disease, to those presenting primarily with motor dysfunction such as Parkinson's disease and Amyotrophic Lateral Sclerosis [2]. A feature common to all of these disorders is a dearth of therapeutic agents that modify the underlying disease process, with current therapies almost exclusively targeting symptoms rather than aetiology [3,4]. As such, developing a deeper understanding of the underlying molecular deficits that drive neuronal cell death is absolutely critical, as gaining insight into these events will open up novel routes to disease-modifying treatments [5]. Building on advances in our comprehension of the underlying genetics of neurodegenerative disease, in particular the identification of Mendelian forms of neurodegeneration and common risk variants deriving from genome-wide association studies, disruption of cellular processes involved in the trafficking and function of vesicles within the cell has emerged as a convergent pathway across a broad spectrum of neurodegenerative diseases [6].

## Vesicular function in the central nervous system

The packaging and trafficking of membrane-bound vesicles is critical for the normal function of eukaryotic cells, responsible for ensuring that cellular components are located in the right place, and at the right time, to carry out physiological roles [7]. The correct regulation of vesicle function and location is required across the full spectrum of eukaryotic cell biology, from energy generation at the mitochondrion through to the regulation of gene expression in the nucleus. This is especially true of the cells of the central nervous system, spanning both neurons and glial cell populations. Neuronal cells, with their extended axons requiring long-range vesicle transport, and the critical role played by synaptic vesicles in neurotransmission, are particularly sensitive to disruptions in vesicle trafficking and function [8]. Coupled to their unique energy demands and the low endogenous rate of neurogenesis, it does not require a great deal of additional stress deriving from vesicular dysfunction to push neurons into a degenerative spiral. More recently, there has been an increasing interest in the role of glial cells, such as astrocytes and microglia, in the neurodegenerative process – with the regulation of vesicular trafficking and function being likewise important for the normal function of these cells [9].

## Neurogenetics of vesicular dysfunction

The neurogenetics of inherited forms of neurodegeneration, and more recently population-based analysis of genetic risk for neurodegenerative disorders through genome-wide association studies, have been a key source of evidence linking disruption of vesicular biology to neuronal cell death [10]. A number of genes that are either mutated in familial neurodegenerative disease or have been identified as risk loci for these disorders have normal functions linked to vesicular biology. Intriguingly, this is true across a range of disorders, including disorders of cognition such as Alzheimer's disease and frontotemporal dementia, and those where the predominant impact is upon movement such as Parkinson's disease and the hereditary spastic paraplegias. These disorders are differentiated by their clinical presentations, but also by the distribution of neurodegeneration within the brain and by the neuropathological hallmarks associated with disease (most notably proteinaceous inclusions, such as amyloid plaques and neurofibrillary tangles in the Alzheimer's brain, and Lewy bodies in Parkinson's) [11].

## Pathways and processes

**Figure 1:**
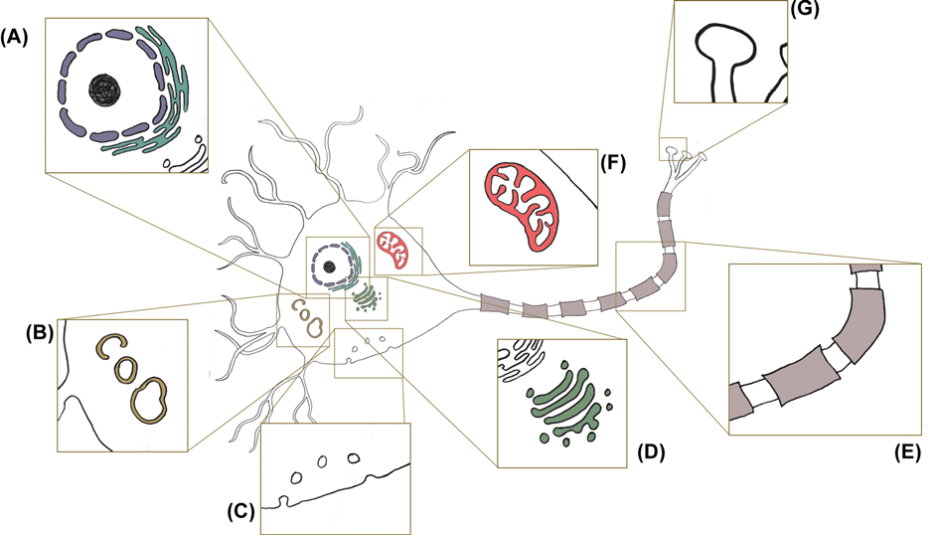
Cellular pathways connecting vesicle biology, trafficking and neurodegeneration, illustrated in a (not to scale) stereotypical neuron (**A**) Nuclear transport. (**B**) Regulation of autophagic pathways and lysosomal biology. (**C**) Endo- and exo-cytic pathways. (**D**) Endosomal vesicle trafficking among the Golgi apparatus, endoplasmic reticulum and the cytoplasm. (**E**) Axonal transport. (**F**) Mitochondrial dynamics and damage response. (**G**) Synaptic function.

Focusing on neuronal cell biology, several critical cellular pathways emerge from the neurogenetics of vesicular dysfunction in neurodegenerative disease. These are summarised in Figure 1, and span highly conserved processes operating in the soma such as the regulation of mitochondrial biology and waste disposal via the lysosomes, through the trafficking of vesicle up and down the axon, to the regulation of synaptic vesicle function.

To discuss and debate these pathways exhaustively would require a textbook, and so in the interests of brevity three case studies, taken from a range of neurodegenerative diseases, will be used to illustrate how a mechanistic understanding of vesicular pathways to progressive neuronal loss is being established. It is also worth pausing to note that, as alluded to above, it is increasingly clear that there is a significant contribution of glial cell function to the neurodegenerative process and that a neuronal centric paradigm for these disorders is a huge oversimplification of what is occurring in the brains of people undergoing neurodegeneration. Unfortunately, how glial biology is disrupted at a pathway level in neurodegenerative diseases remains under explored, and incompletely understood.

### Molecular vignette 1: PINK1, Parkin, mitophagy and the substantia nigra

Mitophagy is a specialised form of macroautophagy, a process that is conserved throughout the *eukarya* as a means to degrade and recycle cellular waste [12]. Macroautophagy is a multistage process, involving the formation of a phagophore (an open, double-membraned vesicle) which then engulfs material targeted for degradation. The encapsulated material is then trafficked to the lysosomes where the autophagosome fuses with lysosomal vesicles and its contents are broken down. The molecular mechanisms governing the selective targeting and degradation of dysfunctional mitochondria first came to light through the discovery of mutations in rare autosomal recessive forms of juvenile parkinsonism, with loss-of-function mutations in the *PRKN* gene on chromosome 6 identified in several Japanese kindreds in 1998 and subsequently mutations in the *PINK1* gene on chromosome 1 reported in 2004 [13,14]. Mutations in both genes resulted in a similar clinical presentation and disease course, with early onset of symptoms during childhood or adolescence and an extended, motor symptom dominant presentation [15]. The cellular functions of Parkin, an E3 ubiquitin ligase, and PTEN-induced kinase 1 (PINK1), a serine/threonine kinase, were linked together by a series of elegant studies in the fruit fly, *Drosophila melanogaster*, demonstrating that they acted in convergent pathway to manage mitochondrial health [16,17]. Over the course of the last two decades, and through the work of many laboratories, there is now a detailed understanding of how these proteins act to coordinate the targeted degradation of dysfunctional mitochondria [18]. PINK1 is activated by the presence of damaged mitochondria (this can be induced artificially by the use of chemical stresses such as Carbonyl cyanide *m*-chlorophenyl hydrazone, which decouples the mitochondrial respiratory chain), and is stabilised at the outer mitochondrial membrane [19]. PINK1 then recruits and phosphorylates ubiquitin and then Parkin, leading to the formation of ubiquitin chains on the surface of the damaged mitochondrion. This, in turn, leads to the recruitment of the proteins involved in macroautophagy and the formation of a phagophore in proximity to the mitochondrion, eventually leading to its engulfment by an autophagosome, trafficking to the lysosomes, and degradation. The consequence of losing the function of PINK1 or Parkin in the human brain is localised degeneration of dopaminergic neurons in the *substantia nigra*, demonstrating a key role for a very specific aspect of vesicular tagging and targeting in neurodegeneration [20]. It is of note that this is distinct from specific dysfunction of vesicle trafficking, highlighting that what is in vesicles can be just as important as where those vesicles are. There is also a clear link between the PINK1/Parkin pathway and broader dysfunction of endolysosomal biology, a cellular pathway of increasing interest and importance across the whole spectrum of neurodegenerative diseases [21]. Equally intriguing is the accumulating evidence for a convergence of glial mitophagy and neurodegeneration [22,23].

### Molecular vignette 2: axonal transport and degeneration of motor neurons

Motor neurons, connecting the brain to effector muscles, possess the longest axons in the human nervous system [24]. As such, they are exquisitely dependent upon the anterograde and retrograde trafficking of material, including vesicular structures, between the soma and synaptic terminals – to an extent even greater than is normally the case for neurons [25]. The progressive loss of motor neurons is a defining pathological characteristic for a heterogeneous group of neurological disorders including, amongst others, amyotrophic lateral sclerosis, spinal muscular atrophy and the hereditary spastic paraplegias [26–28]. Across this spectrum of disease, mutations in a large number of genes have been identified with one of the many areas highlighted being proteins with specific functions in vesicular trafficking – prominent examples being the *SPAST* gene involved in the regulation of microtubule dynamics and the *ATL1* gene, both of which have been heavily implicated in vesicle trafficking centred around the endoplasmic reticulum [29]. Mutations in four genes with direct roles in axonal transport, *DCTN1, TUBA4A, KIF1A* and *KIF5A*, have been identified in familial forms of amyotrophic lateral sclerosis (*DCTN1, TUBA4A, KIF5A*) and hereditary spastic paraplegia (*KIF1A* and *KIF5A*) – with further mutations in *DYNC1H1* (coding for the dynein heavy chain) linked to spinal muscular atrophy and Charcot-Marie-Tooth disease [30–35]. Mutations in *DCTN1*, coding for Dynactin 1/p150^glued^, directly impact on the function of the dynactin complex (a large protein complex that works with dynein to transport material along microtubules) [31]. In cellular and animal models, these mutations cause axonal trafficking deficits – with a specific impact on the trafficking of vesicles [36]. This results in the accumulation of vesicles in axons, axononal swelling, disrupted transport and degeneration. For *KIF1A* and *KIF5A*, mutations causing hereditary spastic paraplegia – associated with the dysfunction and degeneration of upper motor neurons – and amyotrophic lateral sclerosis alter the function of kinesins, motor proteins that shepherd vesicles up and down the microtubular routes along the axon. Although the molecular consequences of these mutations have not been characterised in detail, data from *KIF5A* knockout mice and *in vitro* analyses indicating that removal of kinesin heavy chain leads to axonal transport dysfunction and neurodegeneration, implies that the human disease causing mutations are loss-of-function [37,38]. Further experiments assessing *KIF1A* mutations are consistent with a decrease in function [39]. For *TUBA4A*, coding for α tubulin 4A, rare coding variants and nonsense mutations have been demonstrated to alter the ability of this protein to integrate and form microtubules. The human genome possesses a range of tubulin genes, providing a degree of redundancy in the system for these critical cytoskeletal proteins [40]. It is plausible that mutations in *TUBA4A* are enriched in amyotrophic lateral sclerosis due to the heightened requirements for axonal transport in motor neurons and a specific role for α tubulin 4A in these cells, however this is yet to be addressed experimentally in any depth.

Dysfunction of the dynein heavy chain, encoded by *DYNC1H1*, was first implicated in motor neuron survival through the characterisation of a mouse line with a motor phenotype, dubbed the legs-at-odd-angles or *loa* mouse [41]. This was revealed to have a coding mutation in the *dync1h1* gene causing the phenotype. Subsequent investigations revealed mutations in the human *DYNC1H1* gene casusing a range of motor neuron disease phenotypes, and investigations using mouse, cellular and *in vitro* models have revealed specific deficits in the trafficking of vesicles [42,43]. Taken together, these examples provide clear evidence of defined deficits in axonal transport of vesicles across the broad spectrum of diseases of motor neurons. As noted at the start of this vignette, in many ways this should not be a surprise given the axonal characteristics of motor neurons and their specific sensibilities, however understanding the molecular details of these deficits provides both a window on which genes involved in axonal trafficking are differentially relied upon by motor neurons, as well as potential routes to developing therapies to ameriorate these deficits.

### Molecular vignette 3: the DNAJ proteins, endocytic vesicle trafficking and parkinsonism

Endosomal trafficking at the interface of a cell and the outside world, both within the cell and the process of moving material into (endocytosis) and out of (exocytosis) cells, is a requirement for continued cell viability and is central to a host of cellular functions [44]. This is certainly true of cells in the central nervous system, with specific uptake mechanisms playing important roles in neuronal function [45]. As would be expected for a process that is observed in almost all cells, and across a wide range of cellular roles, there are a number of specialised forms of endocytosis [46]. One particular type of endocytosis has come to prominence in context of neurodegeneration is clathrin-mediated endocytosis. Clathrin-mediated endocytosis is a specialised form of endocytosis that involves the invagination of the plasma membrane followed by the formation of a protein cage consisting of clathrin assemblies around the emerging endocytic vesicle. This then buds off into the cytosol, where the clathrin cage is eventually deconstructed and the vesicular contents sent on their way [47]. In the context of neurodegeneration, mutations in DNAJ proteins, a family of chaperones with multiple inputs into the life cycle of a clathrin-coated vesicle, have been identified in a range of disorders presenting with parkinsonism, as well as lysosomal storage disorders (a group of diseases that share a number of aetiological features with parkinsonism) [48]. Autosomal dominant mutations in the *DNAJC5* gene, encoding cysteine string protein α, cause a hereditary form of neuronal ceroid lipofuscinosis – a form of lysosomal storage disorder characterised by the accumulation of lipofuscin within the cells of the central nervous system [49]. This results in neurodegeneration and a complex clinical phenotype, including parkinsonism in some patients. Cysteine string protein α has an important role in ensuring the correct folding and function of SNARE protein complexes, protein assemblies that are required for correct vesicle formation at the cell surface, and alteration of this function with brain cells disrupts endocytic processes. *DNAJC6* (encoding auxilin) is a brain expressed gene that is required for the uncoating of clathrin-coated vesicles, coordinating chaperone-mediated disassembly by HSC70. Recessive mutations in *DNACJ6* cause a complex young onset form of neurodegeneration, involving parkinsonism as part of its clinical presentation [50,51]. Although the mutations vary in terms of genetic mechanism (spanning clear loss of gene expression through to single coding variants), it is probable that the deficit driving disease is a reduction in auxilin function, thereby disrupting the uncoating and further trafficking of clathrin-coated vesicles [52]. Intriguingly, *GAK*, which codes for the auxilin paralog GAK, is a candidate gene for a Parkinson's disease risk locus on chromosome 4 [53]. In addition to coordinating chaperone activity through its J domain, GAK also possesses a protein kinase domain, and is uqiquitously expressed [54,55]. Although *GAK* has not yet been confirmed as the gene driving association at this locus, this raises the possibility that there is a spectrum of risk associated with clathrin uncoating and Parkinson's, with loss of function in *DNAJC6* causing a Mendelian form of parkinsonism, and more common (and subtle) variation in *GAK* associated with heightened risk of Parkinson's disease. Finally, autosomal dominant coding mutations in *DNAJC13*, coding for RME-8, have been reported in monogenic forms of Parkinson's disease [56]. RME-8 has a similar role to auxilin, with its activity centred around coordinating trafficking of vesicles in the endosomal system and trans-Golgi network. It is of note that there are conflicting reports relating to the pathogenicity of *DNAJC13* mutations, highlighting the need for careful appraisal of genetic risk for human disease, however the biology is certainly intriguing given the other DNAJ proteins involved in neurodegeneration [57].

## Therapeutic targeting of vesicular dysfunction

A major motivation for investigating the molecular basis of neurodegenerative disease is to facilitate the development of novel therapies for these devastating disorders. How, then, can our growing comprehension of vesicular dysfunction in neurodegenerative diseases aid this process? Drug development for the central nervous system has proven to be an extremely challenging endeavour, with a high failure rate and (to date) very few success stories [58]. One of the challenges facing these efforts is the relatively paucity of drug targets to be investigated, and so the discovery of novel – and potentially tractable – targets associated with membrane biology is of great value to the research community. There has been considerable interest in targeting vesicle trafficking in human diseases for a number of years, with tools developed for a number of aspects of intracellular trafficking [59,60]. With relevance to some of the pathways discussed above, endocytic biology and lysosomal function are examples where substantial efforts have been expended [61,62]. A major obstacle to these efforts is the ubiquitous importance of such trafficking and function, with critical roles across a wide range of organs and physiological processes. Achieving specificity in the central nervous system, and avoiding potentially deleterious on target impacts outside of the brain, remains a major challenge.

## Conclusions

The rapid expansion of genetic analysis for neurodegenerative disease has resulted in an abundance of genes implicated in the aetiology of disorders such as Alzheimer's disease and Parkinson's disease. By studying the normal function of these genes, as well as examining the consequences of mutations and concomitant dysfunction, we can gain some insights into the cellular processes and pathways that connect variation in these genes to dysfunction of vesicle trafficking and biology in the central nervous system. The examples discussed above come from disparate forms of neurodegenerative disease, and are pertinent to discrete areas of vesicular biology, but it is striking that the location and contents of vesicles (often in the context of protein aggregation and degradation) is a common theme across these disorders. Whether this is emblematic of convergent biology in these disorders, or a form of functional phenocopy, remains a matter of great debate.

Key questions remain – most notably why do mutations in genes that code for proteins that appear to have ubiquitous function cause specific degeneration of discrete populations of neuronal cells? This is a question that echoes across our current understanding of neurodegeneration, where the basis for selective vulnerability is, at best, unclear [63]. It is indisputable, however, that further investigation and insights into the role of vesicle trafficking dysfunction in the central nervous system will be of great value to future efforts to understand and target neurodegenerative disease [6]. With the application of new technologies and the onward march of human genetics providing ever greater resolution to the genetic architecture and biological basis for these disorders, this is clearly an area to watch.

## Further reading

Clearly, an essay such as this can provide only the briefest of summaries of what is an extensive area of neurodegenerative research. Fortunately there are a number of excellent reviews addressing vesicular biology in neurodegeneration in great detail, a number of which are cited above. The following, picking up on the case studies included in this essay, are particularly recommended:

McWilliams T.G. and Muqit M.M. (2017) PINK1 and Parkin: emerging themes in mitochondrial homeostasis. *Curr. Opin. Cell. Biol.*, 45, 83–91.
Sleigh J.N., Rossor A.M., Fellows A.D., Tosolini A.P. and Schiavo G. (2019) Axonal transport and neurological disease. *Nat. Rev. Neurol.*, 15, 691–703.
Roosen D.A., Blauwendraat C., Cookson M.R. and Lewis P.A. (2019) DNAJC proteins and pathways to parkinsonism. *FEBS J.*, 286, 3080–3089.

## Summary

- Genes identified in Mendelian forms of neurodegeneration and through genome-wide association have highlighted vesicle trafficking as an important area of cell biology in these disorders.
- Cellular characterisation of these genes have revealed deficits in specific cellular trafficking events, including mitophagy, axonal trafficking and endocytosis.
- The biological basis for these deficits are an area of particular interest with regard to the development of novel therapeutics for neurodegenerative disorders.

## Open Access

Open access for this article was enabled by the participation of University College London in an all-inclusive *Read & Publish* pilot with Portland Press and the Biochemical Society under a transformative agreement with JISC.
